# ‘If we would change things outside we wouldn’t even need to go in…’ supporting recovery via community‐based actions: A focus group study on psychiatric rehospitalization

**DOI:** 10.1111/hex.13125

**Published:** 2020-09-09

**Authors:** Johanna Cresswell‐Smith, Valeria Donisi, Laura Rabbi, Raluca Sfetcu, Lilijana Šprah, Christa Straßmayr, Kristian Wahlbeck, Marian Ådnanes

**Affiliations:** ^1^ Finnish Institute for Health and Welfare (THL) Mental Health Unit Helsinki Finland; ^2^ Department of Neurosciences, Biomedicine and Movement Sciences University of Verona Verona Italy; ^3^ National School of Public Health, Management and Professional Development Bucharest Romania; ^4^ Faculty of Psychology and Educational Sciences SHU Bucharest Bucharest Romania; ^5^ Research Centre of the Slovenian Academy of Sciences and Arts Sociomedical Institute Ljubljana Slovenia; ^6^ IMEHPS.research – Forschungsinstitut für Sozialpsychiatrie Vienna Austria; ^7^ Department of Health Research SINTEF Digital Trondheim Norway

**Keywords:** mental health services, patient‐centred approaches, psychiatric rehospitalization, qualitative research, recovery, social determinants of mental health

## Abstract

**Background:**

Psychiatric rehospitalization is a complex phenomenon in need of more person‐centred approaches. The current paper aimed to explore how community‐based actions and daily life influence mental health and rehospitalization.

**Design, setting and participants:**

The qualitative study included focus group data from six European countries including 59 participants. Data were thematically analysed following an inductive approach deriving themes and subthemes in relation to facilitators and barriers to mental health.

**Results:**

Barriers consisted of subthemes (financial difficulty, challenging family circumstances and stigma), and facilitators consisted of three subthemes (complementing services, signposting and recovery). The recovery subtheme consisted of a further five categories (family and friends, work and recreation, hope, using mental health experience and meaning).

**Discussion:**

Barriers to mental health largely related to social determinants of mental health, which may also have implications for psychiatric rehospitalization. Facilitators included community‐based actions and aspects of daily life with ties to personal recovery. By articulating the value of these facilitators, we highlight benefits of a person‐centred and recovery‐focused approach also within the context of psychiatric rehospitalization.

**Conclusions:**

This paper portrays how person‐centred approaches and day‐to‐day community actions may impact psychiatric rehospitalization via barriers and facilitators, acknowledging the social determinants of mental health and personal recovery.

**Patient or public contribution:**

The current study included participants with experience of psychiatric rehospitalization from six different European countries. Furthermore, transcripts were read by several of the focus group participants, and a service user representative participated in the entire research process in the original study.

## 1 BACKGROUND

There is a growing interest in how the social, economic and physical environments may support mental health,^1^, ^2^, ^3^, ^4^, ^5^ with contemporary approaches placing increased focus on the social determinants and the importance providing opportunities for meaningful activities, reducing social exclusion and enhancing community connectedness.^6^, ^7^, ^8^, ^9^ A well‐cited definition by the World Health Organisation (WHO) defines mental health as 'A state of well‐being in which every individual realises his or her own potential, can cope with the normal stresses of life, can work productively and fruitfully, and is able to make a contribution to her or his community',^10^ (p.1) underlining the importance of peoples’ everyday actions on mental health.

Mental health can therefore be promoted both by health‐care services and by community‐based initiatives, which foster positive mental health, prevent mental disorders and should be included across all policy sectors.^11^, ^12^, ^13^ This approach is also endorsed by the WHO Optimal Mix of Services for Mental Health, which emphasizes the need for easily available and balanced mental health services,^14^ while placing considerable emphasis on the value of community and self‐care actions.^15^ Psychiatric rehospitalization is a complex phenomenon that has been reported to hamper the recovery process^16^ and be unfavourable in terms of quality and cost of health care.^17^ Psychiatric rehospitalization is a good example where comprehensive approaches may be beneficial.^17^, ^18^, ^19^, ^20^, ^21^, ^22^ These approaches may also be incorporated prior to hospital discharge, for example ensuring access to meaningful activities, and engaging in signposting and planning for community life during the hospital stay.^23^, ^24^, ^25^ Such approaches reflect a person‐centred ethos where individuals' unique experiences are attended to in a holistic manner and highlight the need for organizing services, organizations, families and communities accordingly.^26^, ^27^

Although a clear definition of person‐centred approaches has not yet been formalized,^28^ it generally reflects health‐care practices where the patients’ perspective and a focus on patient‐reported outcomes are prioritized.^29^, ^30^ Person‐centred approaches may include recovery‐based thinking, which has its roots in service user–led contexts and has gained momentum also within mental health services.^31^ The recovery model redefines how we view mental health difficulties, nurturing empowerment and participation in society.^32^, ^33^ Personal recovery can be thought of as a highly subjective experience around goals, relationships and skills that support a positive life with or without on‐going mental health difficulties.^34^ Although not universally defined, the model acknowledges that mental health treatment may at times be necessary, but views it as one building block in the recovery process, which should also support 'everyday solutions to everyday problems'(Slade 2012).^35^, ^36^

The aim of the current paper was to explore how attention to community‐based actions may support person‐centred approaches in relation to psychiatric rehospitalization using qualitative data from the Comparative Effectiveness Research on Psychiatric Hospitalisation by Record Linkage of Large Administrative Data Sets (CEPHOS‐LINK) project (www.cephos-link.org). Previous papers from the project report on psychiatric rehospitalization using quantitative register‐based methodology^18^ and explore meanings and experiences of the phenomenon qualitatively both in general terms^24^ and more specifically how it can be avoided.^23^ The current paper furthers this line of enquiry via a secondary analysis looking at how participants relate community‐based actions and day‐to‐day activities to their mental health. By articulating what actions individuals with experience of psychiatric rehospitalization themselves deem useful for their mental health, we not only illuminate these actions but also may promote the development of person‐centred approaches in relation to psychiatric rehospitalization.

## 2 METHODS

The CEPHOS‐LINK project studied psychiatric rehospitalization quantitatively and qualitatively in six different countries: Austria (At), Finland (Fin), Italy (It), Norway (Nor), Romania (Rom) and Slovenia (Sl). Ethical approval was sought from leading ethical committees in all participating countries, and informed consent was given by all participants who participated in the qualitative study. A focus group methodology was employed in order to gain insight into lived experience of psychiatric rehospitalization. The reasoning behind this approach related to its suitability for generating insights into experiences, views and meanings through the process of shared discussion derived from both the individual and the groups.^37^ Furthermore, focus groups allow for exploring responses to questions not only in relation to the interview guide, but also in terms of digressions and secondary topics emerging from the process suitable for further thematic analyses.^38^

### 2.1 Data collection and sample

The qualitative data set from the CEPHOS‐LINK project consisted of transcriptions from a total of nine focus groups including 59 service users from the six participating countries: Italy (9 participants), Norway (10 participants), Austria (12 participants), Finland (6 participants), Slovenia (14 participants) and Romania (8 participants). Focus groups were performed by project researchers along common methodological principles,^39^ using a standardized, semi‐structured interview guide. The interview guide was based on four questions asking participants to talk about 1) how it felt to be hospitalized (positive and negative experiences), 2) how it felt to be rehospitalized, 3) aspects important for avoiding rehospitalization and 4) whether the experience is something which participants are open about when discussing with others.^24^

Participants were recruited by convenience sampling via mental health NGOs and/or day centres in each country. The recruitment process was performed by a member of staff at the NGO or day centre in person or via newsletters, home pages or telephone. In Romania and Austria, recruitment and participation took place in capital cities. In Finland and Slovenia, participants were recruited from all over the country, although focus groups were held in capital cities. In Norway, focus groups were conducted in the third biggest city, and in Italy, they took place in a town in the north‐east. Focus groups were led by project researchers with help from assistant moderator(s), lasting between 60 and 90 minutes. Although relatively few focus groups were held in each country, themes became consistent after approximately eight focus groups, indicating a high probability that the study had grasped all relevant aspects.

### 2.2 Data analysis

All focus groups were recorded, and results were transcribed verbatim, translated into English and imported into the NVivo 12 Pro for Windows qualitative software program.^40^ Previous analyses explored questions around how it felt to be hospitalized and how it can be avoided.^23^, ^24^ The current paper is a secondary analysis of the data in response to learning from previous analyses where authors noted that participants often made mention to actions that were located in the community. Data across all interview questions was re‐analysed thematically in two iterations based on principles of systematic text condensation.^41^, ^42^

In the first iteration, the full data set was read several times and analysed following an inductive approach deriving themes and concepts relating to aspects of daily life, which impact mental health. This approach only included aspects outside of the realms of mental health treatment, and content relating to mental health treatment; for example, inpatient or outpatient care (ie visits to psychiatrists, psychologists or primary care professionals) was excluded. Broader comments interpreted as linking everyday life to hospital care were included, for example when participants reflected on personal or community strengths, which could have been beneficial to recognize during the inpatient stay.

The concepts were initially coded into two broader themes reflecting aspects that build up or challenge mental health. These two themes were then further coded into six subthemes. As one of these subthemes encompassed a large number of codes, it was analysed into a further level of five categories. All themes were grounded in the text throughout the analysis.

The process was coordinated by the lead author in consultation with all co‐authors, with representation from all included countries.

## 3 RESULTS

Focus group participants consisted of a total of 59 service users with differing psychiatric diagnoses, who had experienced more than one psychiatric hospitalization and been in contact with mental health services for at least one year at the time of the focus groups. Participants' ages ranged from 25 to 65 years, 61% were female, and almost half of them (48%) lived alone. Most of the participants reported several psychiatric diagnoses: 42% with psychotic disorders, 36% with bipolar disorder, 25% with a depressive disorder and 13% with anxiety disorder. Over 25% had completed a lower or higher degree at university or college, and 10% had completed university or college examinations but did not have a degree. Furthermore, 39% completed secondary/high school as their highest education, and 25% had only completed primary school.

### 3.1 Barriers and facilitators

**FIGURE 1:**
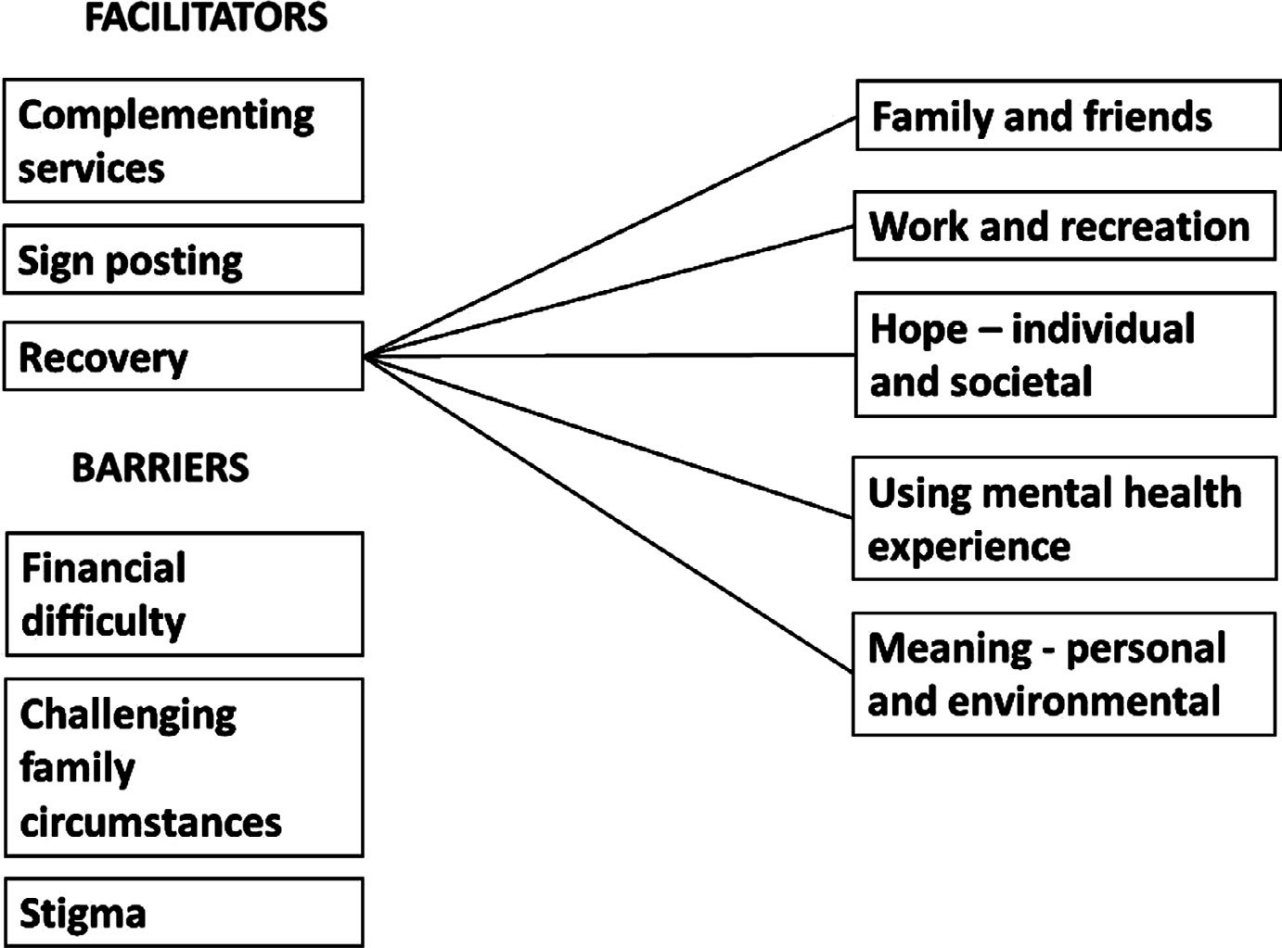
Barriers and facilitating factors influencing mental health and psychiatric rehospitalization

Thematic analysis resulted in a set of six themes and five subthemes, depicting barriers and facilitators to mental health, as illustrated in Figure 1.

Participants described many barriers to mental health including financial difficulty, challenging family circumstances and stigma with the potential for contributing to psychiatric rehospitalization as illustrated by the citation used in the title.

[Sl1/1] … I would change a lot of things outside and inside. I mean, inside as well. But if we would change things outside we wouldn’t even need to go in.

Barriers often related to specific circumstances in life and the social determinants of mental health. Financial difficulties were depicted by participants in relation to problems meeting basic needs in life, and feeling forced to choose between basic needs due to limited finances. Financial instability was also related to an increased risk of rehospitalization.

[It1/8] …I do live alone, my mother is paying for everything I need as I do not have a job, so it’s quite logical that if I do not have a job, then I have some concerns, and then it’s quite obvious that everything sums up and in the end I have to be hospitalised. But if our psychiatrist or psychologist could help us finding a job with [a supported employment organisation] or something else, in order to be able to get up and go on, even if you don’t get so much money….but you have that help, you have it. Otherwise you’re nothing…and it’s obvious then that you can’t stand it, you fall and you have to be hospitalised. It would be much better if there was more concreteness…

Interestingly, psychiatric rehospitalization was also discussed in terms of being a practical option when access to financial resources and other necessities were unavailable. This was depicted as a conscious last resort during particularly difficult circumstances in life. A Norwegian participant recounts how difficulties with drug use and mental health difficulties would lead to rehospitalization.

[Nor2/4] I was so ill I could not look after myself….but at the same time I used the hospitalisations when I had no money for food and that kind of thing. I used the system on my fingertips, at the same time I could not get out of it. I did not get better from that. Sometimes [during hospitalisations] I was very ill, other times I used that side when I had no other choice. I was on the streets in [city] begging for money.

Interpersonal conflicts were also mentioned in the current study. Conflicts were related to conflicts within the family, as well as conflicts in the home community, which were considered as a consequence of mental health difficulties and stigma and prejudice.

[Fin1/1] Well, not with everyone no, you get a feeling quite quickly of who you can talk to, and then you see at home when they change their home town and then you see quite quickly who jumps over to the other side of the road

Participants also described facilitators to mental health in relation to community‐based actions including the following subthemes: complementing services, signposting and recovery. These were typically discussed in the context of NGOs or day centres depending on country. Focus was placed on the nature of what was provided, rather than who provided it as this tended to vary between countries. The recovery subtheme is discussed separately due to its more elaborate nature.

Facilitators to mental health included references to both practical aspects and less tangible ones. A conversation between two Slovenian participants described an NGO as a second home.

[Sl2/8] Well, for me [name of NGO] was a second home. Because I can’t imagine to be at home a whole day long. For me [the authorities] specifically wrote I am not entitled to vocational rehabilitation. So I can’t even work. And without [name of NGO] I don’t know what I would be doing all day. I don’t like to watch TV, I don’t know, I can’t even imagine… Before that I don’t even know what I was doing through the day, I don’t even remember.

[Sl2/14] 'Do you know….speaking about [name of NGO] I am accepted, and if I hadn’t had this environment… At home they have a lot of work for themselves, so if also I would be there, it would be a disaster. I mean, for me because I would be alone in the middle of my family

Actions from community organizations were also described as an important complement to clinical services in terms of both access to support and psychiatric rehospitalization:

[Fin1/2] I have also, where I live, a place where we can come and go and do all sorts together and have peer support groups where we discuss … it has been there for like 11 years that place, and you actually see how people have become stronger and not needed to be taken into hospital

Similarly, an Italian participant relates daily activities as an importance factor of reducing the risk of rehospitalization.

[It1/6] For example, I’m spending some time at [Day Centre] alternating with the outside. In order to maintain the network…I think It’s important to always have a reference point …it’s important to have somebody close to you that knows what to do in critical moments. For example, this morning I’m here, in the afternoon I’ve got something to do, tomorrow afternoon as well. I try to organise for myself something to do…If you have friends and some more things you create something to fill up your days in order to avoid hospitalisation.

Psychoeducation in this context was also discussed in terms of system‐level benefits and in relation to potentially reducing risk of psychiatric rehospitalization.

[Sl1/2] [psychoeducation workshops] … really was a useful thing, we should do things like this more often. You also save money on a systemic level, a person is only hospitalised once because they know how to help themselves.

It is clear however that community‐based actions are not always easy to find. Participants reported benefits of coordinating or ‘signposting’ actions, which facilitate contact with helpful services or organizations in the community.

[At2/2] I think it would make sense, if we already think about it somehow during the hospital stay … what are options for example in the local community, what are the therapy offerings or recreational offerings …that you somehow have a network, that you are rescued, that would be great, that would certainly have helped me.

### 3.2 Recovery

The recovery subtheme contained five categories, family and friends, work and recreation, hope (individual and societal), using mental health experience and meaning (personal and environmental), and was therefore considered separately. Maintaining connections with family, friends and partners and retaining close links to community and daily activity were brought up as important by study participants. Family and friends were predominantly considered to be protective factor for mental health both in terms of having someone to get well for, people to talk to and understand each other, and in terms of help seeking. Genuine and meaningful connections with people were described as important.

[At1/4] What basically just has helped me so much or what I notice is simply that there is somebody [who cares] … that is simply the most important thing.

Participants also discussed this in terms of the importance of maintaining a connection to life outside of hospital during hospital stays, which appeared to instil hope for the future. Keeping connections to the outside world was discussed by a Norwegian participant as an important development.

[Nor2/1] I have never experienced compulsory hospitalisation, so I have all the respect for those who have experienced it, but you are more or less taken out of [your daily] context … To some extent it can be combined [these days] that you get continue their life. Maybe go to work, maybe take care of school or…to maintain what you have… [would be good]…

Connectedness was also overlapped with a sense of meaning. A Slovenian participant discussed the meaning and protective aspects job despite on‐going mental health difficulties. Mental health consequences and loss of meaning were also described in relation to losing employment.

[Sl1/1] …the most important thing for me is my job…. if I see a meaning in it, then I also have this sense of meaning…. I can be stable for a long time….

Hope was described in the current study both on an individual level (with participants talking about how they have managed in the past and therefore are hopeful that they will manage in the future) and on a societal level (how they have been treated in a more positive light than expected, as well as the positive impact of public figures being open about their own mental health difficulties). A Romanian participant recounted how reassurance from an NGO helped to instil hope for the future.

[Rom2/6] I never knew how I am and how… at [name of NGO] they told me that I think very well and they are impressed by what… how I think… and I saved myself only through my way of being… I mean… how I built myself… and I want to help because I live by helping others, I mean I find a way and I try to find a thing that I can… maybe I can live it too, perhaps in the end, … to find something to help another…

Identity was discussed in different contexts. Being viewed as ‘more than a disorder’ was considered key in the current study, overlapping and tapping into both identity and hope. An Austrian participant recounts how she felt like hospital staff viewed her identity in a very one‐sided manner, wishing for other aspects of her daily identity to taken into consideration.

[At2/6] … it simply doesn’t do me good, because they only see me ill and not healthy, I am only a patient there, they then don’t see, how much I have accomplished, the day before I may have managed at home and so on, they don’t see or they also don’t consider it positive, that I was an [names career], that I did the [training for peer support], that I am very active in the aid to refugees now, that is not seen…Instead of saying, great, that you have been managing that over the years, that might help or do good….

Other participants related how they had managed to make use of lived experience of mental health difficulties either in the context of peer support, mentoring or advocacy, offering opportunities for using what has been learnt in a meaningful way, and developing a new sense of self.

[Fin1/2] I talk openly, visit schools and have written about it in the papers you know my story, and I have a blog, for me it has been something that has been positive…

The importance of having meaning in daily life was referred to within the subthemes of personal meaning and environmental meaning. Personal meaning in the current context centred on alleviating boredom (during hospital stay), improving access to meaningful activities, and the importance of spiritual actions both during and following hospital care.

Lack of meaningful activities was recounted by an Austrian participant as a negative aspect of the hospital stay, but mentioned access to spiritual discussions as being very important and meaningful.

[At1/1] … my biggest problem during my hospital stay was always boredom. There is nothing, only a yawning void, there are exceptions of course, but that was the main problem for me, the boredom, and actually no one has time for you. What you can do is, sit in the smokers’ room and watch people smoking.

[At1/1] …the spiritual aspect is also important, … what is the meaning of life and are there acts of God or higher powers, talking about that may also do very good, even if your counterpart says ‘I do not know these things either’….nobody the world over and no human has ever known them, these are things, mysteries, which we all cannot unravel, that also can do good, you know? So, I had conversations with pastors in psychiatry, they were almost best of all, you know

Environmental meaning referred to things in the external environment which participants deemed important for their mental health, for example access to gardening, pets and nature. Participants mentioned this in different contexts both within and outside of hospital.

[Sl2/2] But I really wanted to go home to plant flowers and this gave me strength that I quickly got over and went home

## 4 DISCUSSION

The current paper portrays how community‐based actions and daily life influence mental health, and the risk of rehospitalization via barriers and facilitators. These aspects may be of value when designing more person‐centred approaches within mental health services, in this case, within the context of psychiatric rehospitalization. Barriers to mental health in this study related predominantly to social determinants of mental health. Numerous previous studies have emphasized the mental health impact of social, economic and environmental inequalities and circumstances of life.^43^ Ameliorating the economic situation of individuals, enhancing community connectedness and combating neighbourhood disadvantage and social isolation may improve population's mental health, some of which were mirrored here.^7^ Although these may not always directly relate to psychiatric rehospitalization, they may contribute towards it as discussed in a paper by Duhig et al who reported that stresses and struggles in the community (including accommodation, interpersonal conflict, social isolation and geographic disruption) increase risk of rehospitalization.^44^ This also appears to follow assumptions of the social causation hypothesis, which implies that an individual's experiencing hardship has an increased risk of subsequent mental ill health.^7^, ^45^

Stigma was pinpointed as another barrier to mental health in the current study and which has been echoed in several previous studies.^46^, ^47^ Rebuilding/redefining a positive sense of identity and overcoming stigma has been mentioned in previous studies as an important step towards personal recovery.^22^ Furthermore, reducing societal stigma via community actions and advocacy work has been highlighted as a successful way of improving mental health outcomes, including impacts on psychiatric hospitalization.^48^, ^49^

Linking in aspects of daily life in order to support mental health was highlighted also in a previous literature;^50^ however, it is important to note that this does not happen by itself but requires a conscious effort via relationship building, intersector relationships, training and funding mechanisms.^51^ Incorporating mental health into all sectors via a Mental Health in All Policies (MHiAP) approach could be a useful way to ensure that the social determinants of mental health are considered more systematically.^44^

Facilitators in this study such as the nature and accessibility of community organizations related to actions that had a positive impact on mental health. Access to psychoeducation in such contexts, that is acquiring illness‐specific information including early recognition and management of symptoms, identifying individual stressors, and engaging in mental health promotion, problem‐solving and communication skill training, was described in empowering terms and has also shown promising results in decreasing the risk of psychiatric rehospitalization elsewhere.^52^ Furthermore, previous studies have recounted positive effects of physical, social and creative actions when conducted in a non‐judgemental atmosphere via shared experiences, camaraderie, flexibility and choice.^50^ Participants in the current study described community‐based organizations as feeling like a ‘home away from home’, a sentiment which has been reflected in previous work with workers in this context being described as particularly accessible and approachable.^53^, ^54^, ^55^ This less tangible sense of approachability has also been highlighted in a recent Norwegian study describing circumstances where health professionals conveyed a genuine interest in service users instilling an important sense of being seen and of being valued.^56^

Many of the facilitating subthemes related to personal recovery and were therefore collated into a separate recovery theme. As mentioned in Background section, personal recovery will mean different things to different people, focusing not on cure or absence of mental health difficulties, but on the process of building a life that feels meaningful.^26^, ^57^ This was also underlined by Borg and Davidson, whose qualitative study emphasized the need for a citizen‐led identity, and not one that focuses solely on illness or mental health difficulty.^58^

Maintaining connections with family and friends was predominantly brought up as a positive and protective factor for participant's mental health. This was mentioned in terms of connections to the community during hospital stay and underlined the value upholding links to the outside world such as work and study. Such findings line up with previous literature echoing the importance of maintaining a sense of identity despite mental health difficulty, something which has been articulated as a core aspect of personal recovery.^59^ Reducing social isolation and avoiding feelings of being dismissed or forgotten have also been discussed in this context in previous studies,^60^ and work has been described as an empowering and connecting factor with valuable benefits for recovery.^61^ Retaining worker identity has been reported as an important connector for participation and the recovery.^62^ Recreation has also been pinpointed as an important facet for recovery, with the nature or form of the recreational activity (eg sport, exercise, art, crafts, visits with friends) being secondary to the meanings derived from it.^63^

The concept of hope has been given increased attention within different fields of mental health including conceptions of recovery.^64^ The current study related to not only hope in terms of individual aspects such as managing illness, but also hope from society. A sense of hope was discussed in the current context in terms of new possibilities arising from lived experience of mental health difficulties, and redefining new roles and identities. Peer support work has shown promise in promoting hope and belief in recovery, empowerment and increased self‐esteem, self‐efficacy and self‐management, and supporting social inclusion, engagement and increased social networks in previous literature.^65^, ^66^ Interestingly, a previous study by the CEPHOS‐LINK group found peer support workers a ‘safer’ alternative than talking to health‐care workers as there was no risk of hospitalization in this context.^23^ Further benefits of peer support can be found, with peer mentor programmes having found to relate to fewer rehospitalizations.^67^, ^68^

Meaning is a very personal experience that can be promoted in different ways, participants in the current study mention the importance of spiritual meaning, nature and engaging in activities that feel personally significant. The importance of the everyday environment upon mental well‐being has been reported elsewhere,^69^ as has the influence of eudaimonic well‐being reflecting judgements of meaning and purpose in life.^70^ Meaning can also be supported by incorporating the environmental landscape into day‐to‐day life including agriculture, farms, animals, plants, gardening and the forest that have been found useful for promoting mental and physical health,^71^ something which is also worth attending to in a hospital environment.^72^ However, the authors are not aware of any previous studies relating these aspects to psychiatric rehospitalization per se.^73^, ^74^

Promoting recovery from mental health difficulty can be done in different ways, and aspects from community life can be seen to play an important part in this. Despite considerable steps forward, transforming mental health services towards more recovery orientated has proved challenging, predominantly due to the misconceptions and unclear use of the recovery concept. Slade et al outline seven misuses of the concept of recovery and remind us of the need for partnership working in order to advocate for equitable, holistic and diverse services.^75^ It is important to acknowledge that 'successful' personal recovery does not necessarily equate to zero hospital stays or rehospitalizations. It does however emphasize the need for broader, more comprehensive approaches including self‐care and community resources in an optimal mix of different services for different recovery needs.^76^ Person‐centred approaches may also benefit from increased attention to facilitators such as community‐based actions, as well as barriers including the social determinants of mental health. Community‐based actions may intrinsically follow some of the main presumptions of the recovery model, fostering hope and self‐determination, and promoting social inclusion and human rights, with a stronger focus on daily life and the social determinants of mental health.^77^, ^78^ Delineating these actions is an important endeavour considering that health outcomes of community actions often go unnoticed.^79^ Further knowledge on whether the manner in which community actions are organized could be of interest, specifically in terms of whether administrative and structural factors (such funding models) influence accessibility and results. Exploring the influence of organizational aspects, for instance, whether outcomes are different depending on whether actions are coordinated by civil society, or whether they are organized as part of municipal services, or within mental health services themselves could be a worthwhile topic of future study. Arguably and in a backdrop of funding cuts and austerity measures, teasing out the benefits of community‐based actions is important, not only to justify the need for such services, but also to champion benefits of promoting mental health in day‐to‐day life via more asset‐based approaches.^80^

## 5 STRENGTHS AND LIMITATIONS

A clear strength lies in the rich qualitative data from six European countries and contexts, and its unique approach looking at person‐centred approaches to psychiatric rehospitalization including aspects such as the social determinants of mental health and personal recovery. A more detailed country comparison would have required several focus groups in each country, which was outside of the aim and scope of the CEPHOS‐LINK study. There were however remarkable similarities between countries, participants bring up similar aspects of daily life as supporting their mental health.

Different mental health difficulties and demographic compositions were represented in the study, although the authors do acknowledge the potential for a certain degree of selection bias stemming from participants being recruited via mental health NGOs and day centres. This process may have unintentionally included participants experiencing higher levels of personal recovery with a natural interest in discussing these topics, and who may have had a higher level of education and social functioning, an effect which has been found also in other qualitative studies.^81^

The validity of the data was secured by a mutual study protocol and interview guide including the same multiprofessional researcher team throughout. All authors were involved in all aspects of data gathering, transcripts were read by several of the focus group participants, and a service user representative participated in the entire research process. Data included in the current study were based a semi‐structured interview guide addressing the subject of psychiatric rehospitalization.^24^ The current study employed a secondary analysis of the data intended to further this line of enquiry by exploring how day‐to‐day life was brought up in the context of this interview guide. The current study would have benefitted from more specific questions within interview guide relating more specifically to the research question in the paper. However, this secondary analysis provides interesting insights and is a potential springboard for further study.

Although secondary data analysis may present methodological and ethical difficulties, the current paper can be seen to adhere to the three guiding principles for secondary analysis as articulated in a recent paper by Riggariano and Perry.^82^ The authors have provided a clear and transparent description of the secondary analysis, are very familiar with the parent data methods and context as the same authors have been involved throughout, and the consequence of this secondary analysis has been explored. Furthermore, secondary analysis falls under the same ethical approval as the parent study and previous articles.

Including different European countries contributes to a rich set of data; however, results will undoubtedly reflect the specific characteristics of services available in those regions, and may not represent the entire country. Specific questions on what is was about the community‐based actions services that were deemed beneficial would have allowed for inferences to be made around cross‐country differences.

## 6 CONCLUSION

Person‐centred perspectives are increasingly being incorporated into different areas of health care and can be considered to be a distinctive feature of a recovery‐oriented approach.^27^ This paper provides a valuable perspective on how person‐centred approaches and day‐to‐day actions in the community may impact rehospitalization via facilitators and barriers of mental health. Facilitators to mental health included many elements of personal recovery. Although the definitions, roots and traditions seem to be different, recovery and person‐centred approaches have common features.^27^ Taking the whole person into account lies at the base of both of these approaches, and collaborative action is needed, which acknowledges the multidimensionality of mental health and the impact of the social determinants and community actions.

By taking a broader approach, this paper highlights the relevance of community actions and legitimizes increased attention to day‐to‐day factors. This approach also responds to a call for further research actions supporting a public mental health approach incorporating positive mental health and protective factors.^83^ By incorporating a broader focus and comprehensive approaches, which include community‐based actions in different forms, the current study also endorses a Mental Health in All Policies (MHiAP) approach based on community strengths.^84^

## CONFLICT OF INTEREST

All authors declare that they have no conflict of interest.

## AUTHOR CONTRIBUTION

Cresswell‐Smith, Johanna, Donisi, Valeria and Ådnanes, Marian have made substantial contributions to conception and design, acquisition of data, and analysis and interpretation of data, and been involved in drafting the manuscript and revising it critically for important intellectual content. Rabbi, Laura has made substantial contributions to acquisition of data, and analysis and interpretation of data, and been involved in drafting the manuscript and revising it critically for important intellectual content. Sfetcu, Raluca and Šprah, Lilijana have made substantial contributions to conception and design, acquisition of data, and analysis and interpretation of data, and been involved in revising the manuscript critically for important intellectual content. Straßmayr, Christa has made substantial contributions to conception and design, acquisition of data, and analysis and interpretation of data, and been involved in revising the manuscript critically for important intellectual content. Wahlbeck, Kristian has made substantial contributions to conception and design, and been involved in revising the manuscript critically for important intellectual content and given final approval of the version to be published.

## Data Availability

The data that support the findings of this study are available from the corresponding author upon reasonable request.
